# ENABLING PRIORITIZATION FOR AMYLOID POSITIVITY TESTING WITH A DIGITAL COGNITIVE ASSESSMENT

**DOI:** 10.1093/geroni/igae098.3617

**Published:** 2024-12-31

**Authors:** Ali Jannati, Karl Thompson, Claudio Toro-Serey, Russell Banks, John Showalter, David Bates, Sean Tobyne, Alvaro Pascual-Leone

**Affiliations:** Linus Health, Boston, Massachusetts, United States; Linus Health, Boston, Massachusetts, United States; Linus Health, Boston, Massachusetts, United States; Linus Health, Boston, Massachusetts, United States; Linus Health, Boston, Massachusetts, United States; Linus Health, Boston, Massachusetts, United States; Linus Health, Boston, Massachusetts, United States; Hebrew SeniorLife, Boston, Massachusetts, United States

## Abstract

Primary care providers currently wait for a complaint to initiate a ‘for-cause’ cognitive evaluation and often refer the patient to a specialist. Specialist evaluation then leads to the diagnosis, but treatment occurs late and is unlikely to meaningfully prevent or reduce disability. We hypothesized a brief digital cognitive assessment (DCA), the Digital Clock and Recall (DCR), could concurrently identify CI and predict PET Aβ status, thereby providing an efficient means for timely identification and prioritization of patients for disease-modifying treatments. 930 participants (age 72.0±6.7; 56.8% female; 23% minorities) were classified as cognitively unimpaired, mild cognitive impairment, or probable Alzheimer’s dementia, and 35.1% were Aβ+ on 18F-florbetapir PET scan. DCR-based models were compared with blood-based biomarkers (BBMs) Aβ42/40, pTau-181, and pTau-217, which poorly classified CI (AUCs=0.63, 0.66, 0.72) but accurately classified Aβ status (AUCs=0.81, 0.78, 0.89). DCR accurately classified CI and predicted Aβ status (AUCs=0.85, 0.83). Moreover, combining the DCR with Aβ42/40, pTau-181, and pTau-217 improved their Aβ-status prediction performance to AUCs of 0.882, 0.835, and 0.905, respectively. DCR was superior to BBMs in detecting cognitive impairment and boosted their Aβ-PET prediction ability to levels comparable to CSF biomarkers. We propose a workflow in which implementation of DCAs such as the DCR facilitates early identification of individuals with MCI or early dementia likely due to AD, thanks to the concurrent classification of CI and accurate prediction of Aβ PET status.

